# Undergraduate paramedic students and interpersonal communication development: a scoping review

**DOI:** 10.1007/s10459-022-10134-6

**Published:** 2022-07-19

**Authors:** Jennifer Mangan, John Rae, Judith Anderson, Donovan Jones

**Affiliations:** School of Nursing, Paramedicine and Healthcare Sciences, Charles Sturt University, Bathurst, NSW Australia

**Keywords:** Education, Interpersonal communication, Interprofessional education, Paramedic student, Simulation, Work-integrated learning

## Abstract

The objective of this review is to examine the current literature related to interpersonal communication skill development within undergraduate paramedicine. Interpersonal communication is a vital paramedic skill, with evidence demonstrating it leads to improved patient outcomes and satisfaction and reduces medical errors. Interpersonal communication is a core capability set by paramedicine regulatory bodies, and it is the responsibility of accredited universities to ensure graduates are ready for industry and possess all required skills and attributes. In order to be included in this scoping review, all articles were required to meet a pre-determined ‘population, concept, context’ (PCC) framework. The population was undergraduate paramedic students within the context of their undergraduate paramedicine programs, and the concept was interpersonal communication education/teaching/training. In June 2021, a search was conducted using CINAHL, Medline, Emcare and ERIC. The articles had to be written in English and published between 2011 and 2021 and non-research sources were excluded. 176 articles were identified in this search and after screening for duplicates and relevancy, 15 articles were found to be eligible. The literature highlighted 4 key themes, including graduate perception of ‘work readiness’, and a variety of learning experiences including alternative work integrated learning (WIL), interactions with specific patient groups and professional learning experiences. The literature demonstrated that interpersonal communication skills are fostered through human interactions, WIL and simulation, within undergraduate paramedicine programs. Findings from the literature review indicate that practising communication through human interactions, afford an increase in confidence, awareness of ability and empathy, and an increased awareness of preconceived biases. Intraprofessional and interprofessional simulation teaching methods demonstrate the potential to build students confidence in communication and awareness of what is required to function well in a team.

## Introduction

Paramedics communicate for many reasons, including connecting and engaging with patients in their care, gathering information from patients and people on the scene, imparting professional knowledge regarding treatment and care, and working effectively and collaboratively in a team of other healthcare professionals (HCP) (Ahpra, 2021b). Paramedics are required to be proficient communicators because they interact with a variety of people, including patients across the life spectrum, from a multitude of social and cultural backgrounds, their family members, bystanders, emergency services and other HCPs (Pap & Simpson, 2021). Furthermore, a paramedic’s working environment is unpredictable, busy, noisy and often emotional, which can be barriers to effective communication (Ross, 2021). Effective interpersonal communication can be considered an essential skill for any paramedic and a requirement for safe practice (Ahpra, 2021b)**.**

In its simplest form: ‘’Interpersonal communication is the verbal and non-verbal interaction between two (or sometimes more than two) interdependent people’’ (DeVito, 2015, p. 18). Hargie (2016) adds that communication is also a transaction, with individuals interchangeably taking on the role of both communication sender and receiver, thus influencing the response and reaction of one another. O’Toole (2020) proposes that this transactional conceptualisation of communication applies well to the healthcare context, whereby the HCP and the patient send and receive verbal and non-verbal messages and interpret what they mean. In addition, O’Toole outlines the possible barriers to communication, such as cultural difference, language, age, gender, and life experience, and suggests these barriers need to be recognised and considered in order to have a successful interaction. Ineffective communication, then, can be considered to arise when paramedics do not overcome barriers, or have a poor understanding of effective communication skills and attributes (Willis & Mellor, 2020).

O’Toole (2020) proposes that effective communication requires a mutual understanding, whereby both parties understand the verbal and non-verbal messages being sent, as well as understanding all internal and external factors which could be influencing the responses. For example, if you are communicating with a patient who is scared and in pain, and this is not considered when communicating with them, you may not have a successful interaction. Furthermore, mutual understanding in healthcare requires respect, empathy and trust and it is these attributes that ensure the patient knows they are valued and understood and this, in turn, leads to a positive connection, known as a rapport (O’Toole, 2020). Providing attention, interest and actively listening to the patient further builds this rapport and establishes further trust, which leads to the patient feeling more inclined to interact and collaborate with the HCP (Arnold, 2020). Establishing a good relationship with the patient will then assist with information gathering, which will help the clinician to understand the patients complaint better, make an accurate diagnosis and lead to better decision making by the paramedic (Pap & Simpson, 2021; Ross, 2021). Furthermore, these skills are important in empowering the patient, ensuring the interaction is a positive experience (O’Toole, 2020). Establishing a rapport, being respectful, gaining trust and active listening are all skills a paramedic must possess according to the Australian Health Practitioner Regulation Agency (Ahpra, 2021b)**.**

Effective communication with patients is associated with improved satisfaction and better patient outcomes (Henderson, 2019), whereas poor communication can lead to patient complaints and medical errors (Pilbury & Lethbridge, 2019). More specifically, effective communication leads to a better understanding of the care being provided, and of the healthcare instructions from the clinician, as well as a feeling of reassurance (Henderson, 2019). It is essential for a successfully performing team (Cormack & Scott, 2020), improves patient safety, facilitates more effective care, and increases job satisfaction (Australian Commision on Safety and Quality in Health Care, 2022; Bekkink et al., 2018). It is therefore vital for entry-level paramedics to possess these skills when entering the industry.

Within Australia, there has been a shift in how paramedics are educated. This shift is from a vocational training model to predominantly university-based education (Brooks et al., 2018) and this reflects the level of autonomy and responsibility Australian paramedics now possess (Moritz, 2018). For example, in the state of Victoria between the years of 2001 to 2007, the paramedicine workforce entry-level recruitment went from less than 10% having qualified through a university paramedicine program to 100% (Joyce et al., 2009). Accredited paramedicine programs in Australia are required to establish a course which will ensure its graduates possess the knowledge, skills and attributes required to practise as a paramedic, including being an effective communicator (Ahpra, 2021a, b).

A number of studies have explored the interpersonal communication skills possessed by paramedic students. Ross et al. (2014) examined paramedic students’ perceived communication abilities and found that students generally felt they had good communication skills, especially in areas such as empathy and being supportive. Similarly, Boyle et al. (2011) investigated perceived listening and communication styles of paramedic students and found the student’s preferred styles were characterised as being friendly, and aware and attentive to someone’s feelings. Conversely, Willis et al. (2010) presented data indicating that many of the desired interpersonal attributes, including communication, teamwork, and empathy that should be found in a graduate were in fact not. This is supported by Williams et al. (2016) who identified lower levels of self-reported empathy within paramedic students, when compared to other health professions.

Lazarsfeld-Jensen (2010) emphasised that interpersonal communication skills should not be assumed to be intrinsic to paramedic students, and recommends universities teach interpersonal skills and other ‘non-technical’ skills with a degree of importance equal to clinical skills. Prior to 2012, no research was published regarding interpersonal communication education, within undergraduate paramedicine (Ross, 2012). This gap in the literature demonstrates that communication education has been undervalued within paramedicine in the past and this research intends to examine if this has changed. The aim of this scoping review is to examine current literature regarding the learning and teaching of interpersonal communication within undergraduate paramedicine in the last decade.

## Method

This scoping review followed guidance from the Joanna Biggs Institute (JBI) scoping review methodology, including the development of a priori protocol which was agreed on by all authors (Peters et al., 2021). Scoping reviews afford an exploration of emerging and heterogeneous literature, with the aim of identifying the concepts in that field and informing further research (Peters et al., 2021). Scoping reviews also support the exploration of a broader research question, which may pave the way for future systematic reviews, where a more specific question can be posed (Peters et al., 2020).

### Research aim and question

This literature review aims to examine current literature regarding interpersonal communication learning and teaching within undergraduate paramedicine. The research question therefore is –

What current evidence base exists to inform universities to prepare their undergraduate paramedic students to be competent communicators?

### Eligibility/inclusion criteria

In order to be included in this review, all evidence needed to meet the population, concept, context (PCC) inclusion criteria (Peters et al., 2021). The population to be examined was undergraduate paramedic students and the concept was the teaching and/or learning of interpersonal communication. This must have taken place within the context of an undergraduate paramedicine course. The literature review by Ross (2012) found no evidence of interpersonal communication education within undergraduate paramedicine prior to 2012, so a date range was added to the inclusion criteria in this scoping review meaning only articles published between 2011 and 2021 were to be included. Non-research sources were excluded in this study, as the inclusion of such, can impact the replicability of the research (Adams et al., 2016). Evidence not published in English was also excluded. These criteria were agreed by the whole team prior to the search.

### Search strategy

A search was conducted using the databases Cumulative Index to Nursing and Allied Health Literature (CINAHL), Medline, Emcare and Education Resources Information Centre (ERIC), accessed via the Charles Sturt University (CSU) online library between the 30th of June and the 1st of July 2021. Search terms were chosen based on the PCC framework already established (Table 1).

**Table 1:** Search terms

Key word search	Title only
	Paramedic* OR ambulance OR out-of-hospital OR prehospital OR pre-hospital OR EMT
AND	
Teach* OR student* OR university* OR undergraduate*	Educat* OR train* OR teach* OR student*
AND	
Communication* OR Interpersonal relations OR nonverbal communication OR soft skill OR non-technical	Communication* or “soft skill*” or “non-technical*” or “non-clinical*”
**Additional search limits** – published between 2011 and 2021	

### Evidence selection

**Fig. 1:**
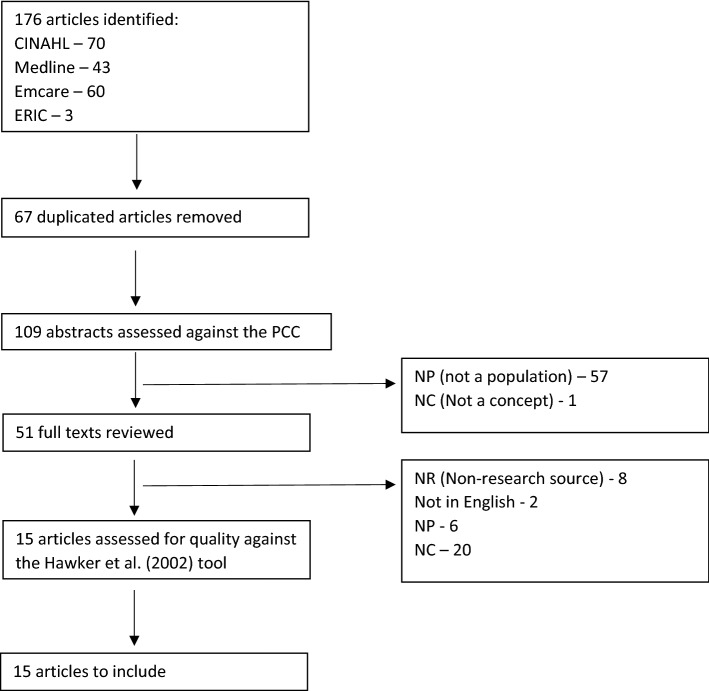
Flowchart of evidence selection

This search identified 176 articles. Sixty-seven of them were duplicates, leaving 109 articles. The abstracts were initially assessed by JS to determine relevance using the PCC framework and 58 were removed. The full texts were then examined by JS and a further 36 were removed, leaving 15 articles (Fig. 1). Reference lists from those 15 articles were scanned to identify any missing articles. Furthermore, each article was searched for in SCOPUS to identify any articles citing them, which may have been relevant. No further articles were identified during this process.

Authors JS and DJ independently assessed the quality of the articles using the Hawker et al. (2002) tool before then assessing the quality together to conclude each articles rating. The Hawker et al. (2002) tool was created due to a need for a robust way of assessing the quality of qualitative and disparate research. As such, due to the variety of methodologies used in the articles presented in this review, the Hawker et al. (2002) tool was appropriate. The tool highlights areas of strengths and weaknesses within each study by investigating 9 keys areas, such as abstract, method or ethics, etc. and rates them on a scale of ‘good’, ‘fair’, ‘poor’ and ‘very poor’ (Appendix 1). All articles included in this review were graded ‘good’. Although not always considered necessary for scoping reviews (Levac et al., 2010), this was conducted to add a degree of rigor to the conclusions drawn from this study (Brien et al., 2010).

### Extracting and charting the evidence

Data were extracted by two authors (JS and DJ) and both were in agreement that the articles were relevant to the PCC framework. The data regarding aims or objectives, sample and setting, method, outcomes, and conclusion and limitations were charted in a table form (Appendix 2). This enabled the evidence to be captured and recorded, while maintaining transparency and limiting errors and bias (Peters et al., 2021).

## Data analysis

The research presented in this review took place within the context of a variety of university settings in multiple countries, including 10 from Australia, 2 from the United Kingdom (UK), 1 from the United States (US) and 1 from Poland. The remaining article was a literature review, which extracted data from the UK and US. The experience of the undergraduate population varied between articles with 7 articles indicating students were either in their 3rd year, final year, or a senior student, 4 articles referred to 2nd year students, 2 articles referred to 1st year students and 1 article did not specify the student’s level of experience. Furthermore, there were a variety of research methods used, with a large proportion using a mixed methods approach. Every article demonstrated examples of the development interpersonal communication; however, this was not a primary aim in all studies.

Extraction of the data in line with the research question identified 4 major themes:

1. Interacting with a specific patient group
2. Professional communication experiences
3. Alternative work-integrated learning placement
4. ‘Work ready’ graduates and undergraduate communication education

## Presentation of results

### Interacting with a specific patient group

In 4 publications, students had the opportunity to interact with various people within the elderly population, including people who are under palliative care and people with dementia. Lucas et al. (2015) and Stratton et al. (2015) utilised a mixed method approach to investigate a 5 day WIL experience in a residential aged care facility (RACF), with final year paramedic students. Both student groups had a variety of structured learning activities and opportunities to interact with aged care residents, many of which had dementia (Lucas et al., 2015; Stratton et al., 2015). This experience improved the student’s knowledge of dementia and the communication barriers it can cause, as well as providing the opportunity for students to develop new strategies to communicate effectively and build a rapport with this particular patient group (Lucas et al., 2015; Stratton et al., 2015). Furthermore, students reported an increased confidence in their communication ability and an increased awareness of how they might transfer these skills into the pre-hospital setting (Lucas et al., 2015; Stratton et al., 2015).

Building a rapport with a specific patient group was also investigated by Ross and Williams (2014). This study was limited by its small, self-selected sample, as well as the fact that the questionnaire used was not psychometrically tested and was based on self-reported data. However, the results were positive, with students reporting a significant improvement in their ability to build a rapport after the students had interacted one on one with an elderly person. Self-reflection demonstrated further improvements in the students’ learning. Results also demonstrated an increase in the students confidence and empathy levels, which was further reflected in the work by Ross et al. (2018). In this study, communication skills improved in all students who practiced communicating with an older person, however those who had more one on one time, developed more empathy towards this patient group.

By providing students with this experience, they have been able to practice and develop key interpersonal skills such as rapport and empathy, as well as develop communication strategies to overcome communication barriers.

### Professional communication experiences

In 3 publications, students experienced professional interactions with other students, in simulated emergency settings. Johnston et al. (2014) and Furseth et al. (2016) used a simulated patient handover, between nursing students and paramedic students, with the aims of overcoming barriers to interprofessional education (IPE) and improving interprofessional communication between the respective students during this crucial interaction (Furseth et al., 2016; Johnston et al., 2014). Johnston et al. (2014) gathered their data in group debriefing sessions and concluded that the students found it valuable to practise handovers and then take part in a debrief alongside other HCP students. Whilst this demonstrated how universities were teaching communication, it was not clear what the students learnt from the experience. Furseth et al. (2016) found high levels of self-confidence after the handover activity, stating that by increasing students’ confidence with a certain task through the means of simulation and other learning activities, students may be more likely to complete these tasks successfully when required to do so in real life. It may have been beneficial for this research to have included a pre-intervention assessment of the students’ confidence level, to better understand the merit of this learning activity. Similarly, Ford et al. (2014) used an intraprofessional learning activity with the aim of developing student’s relational competence, which includes attributes such as being non-judgemental, trustworthy, empathetic, compassionate, and respectful. Students took part in a 3-day simulation activity and data were gathered through field diaries and focus groups (Ford et al., 2014). The students reported a better understanding of teamwork-based communication skills and a recognition of their own personality types when it came to communication (Ford et al., 2014).

In addition to these simulated professional interactions, Ross and Bertucci (2014) investigated student learning after being part of a peer-assisted learning (PAL) program. Paramedic students mentored high school students with an interest in a career in paramedicine and it was found that the program supported the development of interpersonal communication skills (Ross & Bertucci, 2014). The research unfortunately provided no more detail than that, as communication was not the primary outcome of their study. One might speculate that this type of activity may have merit in developing experience in interacting with teenagers and practicing tailoring medical language to a younger person.

Within 3 articles examples were provided of an interpersonal communication learning activity being used which provided students with the opportunity to practice communication. Only within the Ford et al. (2014) publication did authors provide evidence of exactly what communication skills the students were developing. But promisingly, all 4 publications were evidence of universities prioritising communication experiences within undergraduate programs. Simulated professional interactions appear to be enjoyed by the students and allow students to practice working in a team, highlight their own communication skills, and build confidence.

### Alternative work-integrated learning (WIL) placement

Non-ambulance work-integrated learning (WIL) experiences within the community (Ross & Kabidi, 2017; Prakash et al., 2020), and in hospital (Credland, et al, 2020) were examined in 3 publications. Whilst communication was not the primary outcome under investigation, it was an area of development reported by students. On community-based WIL placements, students were exposed to areas of the community they had not interacted with before and this led to the development of empathy and led students to recognising unconscious biases that they may have held prior to the placement (Prakash et al., 2020; Ross & Kabidi, 2017). In 12 interviews, Ross and Kabidi (2017) discovered that this opportunity to practice communication led to an increased confidence and the development of new communication strategies. Similarly, the focus groups conducted by Prakash et al. (2020) highlighted that students immersed in this environment were afforded the opportunity to practice and hone communication skills such a rapport building. Furthermore, semi-structured interviews conducted by Credland et al. (2020) regarding a hospital-based WIL placement, highlighted that students were able to observe and practice various communication strategies with multiple patients, as well as gain experience with inter-disciplinary communication. These publications are examples of how non-ambulance WIL experiences have the potential to develop students’ communication skills. Students were immersed in these new and often unfamiliar environments where their technical paramedic skills were not necessarily required, and students were encouraged to focus on their interpersonal skills as a priority. However, Credland et al. (2020) did provide evidence of students not always feeling welcome or supported whilst taking part in this WIL activity, as well as a lack of understanding from staff as to why paramedic students were there. This could have negative implications for the amount of learning that could take place, if it is not considered when creating non-ambulance WIL.

### ‘Work ready’ graduates and undergraduate communication education

The preceding publications provide examples of interpersonal communication educational experiences and teaching methods. The remaining publications investigate the university experience as a whole and how prepared students were in terms of interpersonal communication when entering the workforce. Włoszczak-Szubzda et al. (2013) analysed the interpersonal communication training within the syllabuses of 20 paramedicine education centres in Poland. Authors found that although communication education was present, there was a lack of job specific communication education, as well as interprofessional communication, with a greater focus on general communication knowledge (Włoszczak-Szubzda et al., 2013). Whereas Twinley (2012) used a mixed methods approach with a self-designed questionnaire in the UK and demonstrated that paramedic students felt they had been adequately taught verbal, non-verbal and written communication skills, but would like more practical experience to further develop their skills. Furthermore, the research showed a trend in the student’s preparedness for WIL placement, whereby they felt less prepared for their first and more prepared for their most recent WIL placement (Twinley, 2012). Whilst the evidence is positive, caution should be exercised as this study was conducted with a small sample and it is unclear in both studies if the evidence is transferable internationally. Undergraduate paramedicine education facilities may differ in course structure, including required ambulance-based WIL hours leading to registration, as well as differences in cultural norms of communication.

An Australian study by O’Brien et al. (2013) investigated 23 final year paramedic students’ perceptions of their work-readiness, using a mixed method survey. Quantitative data demonstrated that students feel ‘somewhat adequately to adequately’ prepared by their degree in terms of general interpersonal skills and communication with HCP’s (O’Brien et al., 2013). Students felt that WIL placements were an activity which could develop their patient communication further, with the authors postulating that paramedicine training could be improved through more WIL opportunities, including more diverse WIL placements, within a variety of healthcare sectors (O’Brien et al., 2013). However, since this publication, Australian paramedicine degrees have become subject to professional accreditation, so information about how prepared students feel to enter the workforce requires updating.

The final article identified by this review is a literature review by Ross (2012). This article demonstrated that before 2012 there was no research conducted concerning interpersonal skill education within undergraduate paramedicine. This article therefore supported the use of a search limitation based on year of publication when conducting the literature search for this scoping review (Ross, 2012).

## Discussion

Providing opportunities to practice interpersonal communication with patients, peers and other disciplines was a prevalent teaching method described in the literature. Practise in this manner appears to build students comfort and confidence in their interpersonal communication skills (Credland et al., 2020; Furseth et al., 2016; Johnston et al., 2014; Lucas et al., 2015; Ross & Bertucci, 2014; Ross & Williams, 2014; Stratton et al., 2015). Through this interaction, students are able to discover their own weaknesses and strengths in their current communication ability (Ford et al., 2014; Prakash et al., 2020; Ross & Kabidi, 2017; Ross et al., 2018) and students consider more opportunities to obtain practical experience as a way to develop their communication ability further (O’Brien et al., 2013; Twinley, 2012). Additionally, in the literature review by Ross (2013), a similar conclusion was drawn, that the use of ‘real patients’ to develop interpersonal communication skills, when introduced early and purposefully within a HCP training, can lead to an increased confidence and ability to build rapport thus supporting the findings from this review. Furthermore the research shows that practising communication with specific patient groups, or with new groups of people that students had not interacted with before, appears to highlight unconscious biases that students may have held and encourages them to question stereotypical ideas they may have felt (Prakash et al., 2020; Ross & Kabidi, 2017; Stratton et al., 2015). These interactions lead to students developing empathy (Lucas et al., 2015; Prakash et al., 2020; Ross & Williams, 2014; Ross et al., 2018). These findings are similar to those found by Perlman et al. (2017), where undergraduate nursing students interacted with a particular patient group during a WIL placement. Perlman, et al. (2017) found that after this experience students were able to recognise and address preconceived biases and discovered the importance of communicating in a meaningful and caring way. Developing these caring attributes such as being kind and non-judgemental are key to being proficient at building rapport (English et al., 2021). Furthermore, Norfolk et al. (2007) reports that empathetic motivation, the ability to empathise, and possessing the communication skills to demonstrate to the other person that someone is empathetic to their situation, are all vital components of establishing rapport. Providing students with the opportunity to interact with specific patient groups, appears to be a beneficial teaching method to foster these attributes (Lucas et al., 2015; O’Brien et al., 2013; Prakash et al., 2020; Ross & Williams, 2014; Ross et al., 2018).

WIL has been explored in a number of the publications presented in this scoping review and appears to be a prevalent teaching method offering students many opportunities to interact with a variety of people (Credland et al., 2020; Lucas et al., 2015; Prakash et al., 2020; Ross & Kabidi, 2017; Stratton et al., 2015).This learning is often assisted by working alongside and with the support of mentors or supervisors (Credland et al., 2020; Lucas et al., 2015; Stratton et al., 2015). For example, research by Stratton et al., (2015) and Lucas et al., (2015) demonstrated that structured learning workshops provided during a WIL placement, alongside interactions with the residents supported student learning. However, only 1 study included in this review provided data regarding the role of the mentor or supervisor during WIL, and the barriers to effective mentorship (Credland et al., 2020). Wongtongkam and Brewster (2017) proposed that a good working relationship between mentor and student is beneficial to the students overall learning while participating in WIL. In addition, Wongtongkam and Brewster (2017) investigated paramedic students’ clinical and non-clinical skill development during an ambulance placement where students have direct support from a mentor and found students improved in all aspects surveyed, which included history taking and questioning, handovers, giving clear communication and building rapport. Michau et al. (2009) discussed that student paramedic skill development is reliant on the mentor encouraging and/or allowing the student to use a skill and ensuring the student feels supervised. This sentiment was reflected in nursing placements too, whereby positive relationships between the nursing students and the professional staff, lead to an increased sense of belonging, motivation, confidence, and growing sense of professional identify, which all support student learning (Levett-Jones et al., 2009). Of course, WIL in a healthcare setting will inevitably expose students to a variety of patients, but supportive mentorship is a vital component to consider when using this as a teaching and learning method. Furthermore, WIL will not expose students to all patient groups, and in order to address these learning gaps, additional learning opportunities on top of WIL need to be considered (Michau et al., 2009).

Simulation was found to be another useful learning method for preparing students to be proficient communicators (Ford et al., 2014; Furseth et al., 2016; Johnston et al., 2014). Simulation offers the students an experience similar to the real thing, where the scope or level of the task can be controlled and with no risk of adverse events to patients (So et al., 2019). Professional interactions, through simulation, appear to lead to an increase in student’s confidence regarding interprofessional communication (Furseth et al., 2016; Johnston et al., 2014), whilst highlighting the importance of team collaboration and effective team communication (Ford et al., 2014). Similar results were found in the review of literature conducted by Granheim et al. (2018), where IPE simulation was found to have a positive effect on communication skills and team collaboration skills. Granheim et al. (2018) highlighted that these IPE experiences encourage student communication development as any weaknesses come to the foreground and can be addressed.

Paramedic to patient communication was simulated using older people in the study by Ross et al. (2018), however it was used to assess the communication ability of the students, not as the learning strategy. Nevertheless, it was likely to be the leading cause of why all the communication scores of all students in the study improved, thus still promoting it as a learning strategy, although not potentially intended as one. Using students as simulated patients, to role-play interpersonal paramedic to patient communication, was not found in the literature, however Twinley (2012) proposed communication development activities such as role-play may have merit if incorporated into paramedicine education as a method of developing key communication attributes. Likewise, developing communication skills required to interact with children or developing skills related to difficult conversations with patients or their loved ones was not found in this review. Anderson et al. (2019) found that difficult conversations relating to terminating resuscitation to be one of the student’s greatest concerns, with the student’s suggesting simulation and role play may be beneficial learning and teaching techniques. Although the use of simulation appears to be beneficial for communication practice, there is no consensus to the level of fidelity required to facilitate interpersonal communication skill development or the potential benefits of more emerging simulation methods within paramedicine such as immersive technology media (Birtill et al., 2021). Exploring the use of technology, Barr and Foster (2017), proposed that the type of immersive technology used in their study, offered a more engaging and authentic learning environment, where a plethora of technical and non-technical skills, including communication were able to be practiced. Furthermore, a literature review by Birtill et al. (2021) exploring the use of immersive technologies in paramedicine concluded that while the role it could play in educating paramedic students is still unclear, it is an area that could benefit from further research drawing from its use and efficacy within other health disciplines.

It appears the overarching method of preparing undergraduate students to be proficient communicators is through experience. Kolb (2014) proposes that for knowledge to be created through experiential learning, the learner is to reflect on the experience and derive new concepts and ideas based on these reflections, to inform the next experience. Many of the studies presented in this review involve an experience and reflection, and in addition, many of the researchers encouraged or facilitated student reflection to gather data, which may have led to additional knowledge and learning by the participants (Ford et al., 2014; Johnston et al., 2014; Ross & Williams, 2014).

### Limitations

Scoping reviews explore the literature over a certain period of time, and there is the potential, more data has become available since this review was conducted, which is a limitation of scoping reviews (Brien et al., 2010). There is the potential, as not all databases were searched, that some articles were missed during this scoping review. Also, paramedicine has many interchangeable names, so there is a potential that the search did not include all paramedicine terms, and this may have afforded some articles being missed. In addition, with the terms focused on undergraduate paramedic students, it should be acknowledged that not all countries have tertiary education as a requirement for paramedic practice. Furthermore, many of the studies used in this review are investigating broader topics as opposed to specifically investigating communication development.

## Conclusion

This scoping review has provided evidence of the teaching methods being used to develop undergraduate paramedic student’s interpersonal communication skills. This review demonstrates that interpersonal communication development is valued by universities, with many offering opportunities for students to practice communication as a priority. Opportunities to practise interpersonal communication whilst taking part in WIL or through interactions with specific patient groups afford increased confidence and ability. These interactions also lead to an increase in empathy and a decrease in preconceived bias, all promoting a better rapport building ability with the patients in their care. In addition, professional simulation activities have the potential to offers student’s the opportunity to build confidence in communication and develop teamwork skills all while in a supportive environment, with the opportunity to reflect and debrief.

Whilst this area of research is still growing, it is promising to see there is an evidence base beginning to emerge to inform universities on how best to support their students. There is strong support for the use of WIL and creating opportunities for students to interact with patient groups to support communication development, but little evidence into the true potential of simulation. Further research is recommended into the role simulation can play in developing student’s interpersonal communication skills. Research is recommended into the various styles of simulation, including role-play, use of standardized patients and immersive technology. This review has demonstrated a growing value and interest in this important area of study.

## Appendix 1

**Table:**

Quality appraisal tool Hawker et al. (2002)G = Good, F = Fair, P = Poor, VP = Very poor	Credland et al. (2020)	Stratton et al. (2015)	Lucas et al. (2015)	Prakash et al. (2020)	Ross and Kabidi (2017)	Włoszczak-Szubzda et al. (2013)	Ross and Bertucci (2014)
Abstract	G	G	G	G	G	G	G
Introduction/aims	G	G	G	G	G	G	G
Method/data	G	G	G	G	G	G	G
Sampling	G	G	G	G	G	G	G
Data analysis	G	G	G	G	G	G	G
Ethics/bias	F	F	F	F	F	VP	F
Results	G	G	G	G	G	G	G
Transferability/generalisability	G	G	G	G	G	F	G
Implications/usefulness	G	G	F	G	F	F	G
Overall appraisal	G	G	G	G	G	G	G

Quality appraisal tool Hawker et al. (2002)G = Good, F = Fair, P = Poor, VP = Very poor	Ross and Williams (2014)	O’Brien et al. (2013)	Ross (2012)	Furseth et al. (2016)	Ross et al. (2018)	Twinley (2012)	Johnston et al. (2014)	Ford et al. (2014)
Abstract	G	G	G	F	G	G	F	G
Introduction/aims	G	G	G	G	G	G	G	G
Method/data	G	G	G	G	G	G	G	G
Sampling	G	G	G	G	G	G	G	F
Data analysis	G	G	G	G	G	G	F	G
Ethics/bias	F	VP	N/A	VP	F	G	VP	F
Results	G	G	G	G	G	G	G	G
Transferability/generalisability	G	G	G	G	G	G	G	F
Implications/usefulness	G	G	G	F	G	G	G	F
Overall appraisal	G	G	G	G	G	G	G	G

## Appendix 2 Charting the evidence

**Table:**

Citation	Aim/objective	Sample and setting	Method	Outcomes	Conclusion and limitations
Ross, L., & Kabidi, S. (2017). Embedding Volunteer Activity into Paramedic Education. *Journal of Allied Health, 46*(3), 192–196	Aimed to explore the value of embedding volunteer activity into undergraduate paramedic education	12, 3rd year paramedic students at an Australian university	Cross-sectional study with one-on-one interviews Thematic analysis of the interview transcripts	Themes relating to the student’s experience – Learning about themselves Learning about the community Skills beneficial for future paramedic practice	Limitations: Small sample size Self-reported data Conclusion: Volunteer activity within a paramedicine program is an effective way to develop required paramedic attributes Awareness and understanding of community groups and needs, interpersonal communication skills, and the ability and confidence to interact effectively with people from a range of population groups were key outcomes
Prakash, S., Brown, S., Murphy, M., & Williams, B. (2020). Paramedic student empathetic attitudes towards homelessness: a mixed methods pilot study. *International Journal of Emergency Services*	The aim was to explore paramedic students’ empathetic attitudes towards homelessness	20, 2nd year paramedic students at an Australian University	Sequential mixed methods study – 2 surveys and a focus group The Medical Condition Regard Scale (MCRS): 11-items are rated on a 6-point Likert scale (15 strongly disagree, 65 strongly agree) (maximum score 66) The Health Professionals’ Attitudes Toward the Homeless Inventory (HPATHI): 19- item which are rated on a 5-point Likert scale (1 = strongly disagree to 5 = strongly agree) Thematic analysis of the focus group by all researchers	MCRS scores—Statistically significant improvement (*p* < 0.05) in scores was observed from pre- data to post- data, with total mean scores of 48.35 (SD ± 8.33) and 51.65 (SD ± 5.56) respectively HPATHI scores—Mean scores for Personal Advocacy were 34.40 (SD ± 4.32) pre-project and 36.70 (SD ± 3.07) post-project and was found to be statistically significant (*p* < 0.0001). Social Advocacy increased post-project with mean scores 24.45 (SD ± 3.50) and 26.00 (SD ± 2.93) respectively. Cynicism were 14.85 (SD ± 14.85) pre-project and 16.15 (SD ± 2.18) post-project Themes in the focus groups: communication, empathy and rapport and a change in perception and attitude	Limitations: Convenience volunteer-based sample Self-selection bias No control over participant’s background Self-reporting, which could lead to participant providing socially desirable answers Focus groups can lead to irrelevant discussion and you need to ensure everyone has a voice Conclusion: The results of this pilot study suggest that paramedic undergraduate students demonstrated an increase in their self-reported empathy levels towards people experiencing homelessness. Increases in measures of empathetic regard, social advocacy and personal advocacy were found
Credland, N., Rodgers, A., Hurwood, M., & McKenzie, J. (2020). Student paramedic perceptions of a non-ambulance practice learning experience. *Nurse Educ Today, 88*, 104,374	Aimed to explore first year student paramedic's experiences of non-ambulance placements	33, 1st year paramedic students at a university in the United Kingdom	A qualitative study with semi-structured interviews using trigger questions Thematic analysis of the interview transcripts	Four key themes that emerged from the transcripts were: Expectations The Patient Journey Communication Mentorship	No limitations stated Conclusion: Students identified learning opportunities whilst on placement, however students felt unsupported at times and reported a lack of insight into the need for a non-ambulance placement from their mentors
Lucas, P. V., McCall, M. J., Eccleston, C., Lea, E., Stratton, B., Annear, M., Crisp, E., Elliott, K.-E., & Robinson, A. (2015). Prioritising the development of paramedic students' interpersonal skills. *Journal of Paramedic Practice*, 7(5), 242–248	The authors explored a placement programme designed to improve students’ interpersonal skills through opportunities to interact with residents with dementia, students from other disciplines and facility staff.’	31 final year paramedic students at an Australian university	Mixed method Pre and post survey and an open-ended feedback meeting Pre-placement surveys—students’ perception of their interpersonal skills, and their attitude towards the placement and the prospect of working with older people. Knowledge of dementia measured using the 21-item Dementia Knowledge Assessment Tool Version 2 (D-KAT2) Post placement surveys—Learning experience, their perceptions of their interpersonal skills, and their knowledge of dementia Thematic analysis of the feedback meeting transcripts	D-KAT2 score—A paired-samples t-test showed a statistically significant increase between the pre- and postplacement mean score Perception of interpersonal skills survey—statistically significant improvement in their interpersonal skills and understanding of other health professions Students attitude survey – Increase in reported enjoyment, confidence and understanding of RACF Themes relating to the experience – Improved understanding of dementia, improved communication skills, increased empathy and understanding	No limitations stated An increased understanding of dementia was identified in the qualitative data and the findings from the quantitative evaluation supported this The findings highlight that participation in the IPL activity involved communication, teamwork, interpersonal, intrapersonal and, in some cases, leadership skills as they collaborated with colleagues from other disciplines in the assessment of residents The final salient outcome of the placement was an increase in students’ understanding of the aged-care sector and an increase in empathy for the residents, as well as staff working in the RACF
Stratton, B., Lea, E., Bramble, M., Eccleston, C., McCall, M., Lucas, P., & Robinson, A. (2015). Residential aged care facility clinical placements for undergraduate paramedic students: An evaluation of the Australian experience. *Australasian Journal of Paramedicine*, 12(2)	Objective is to examine the learning experiences of paramedic students who took part in the first paramedic student clinical placement in residential aged care facilities	21 final year paramedic students at an Australian university	Mixed method Pre and post survey and an open-ended feedback meeting Open-ended feedback meetings using prompting topics based on literature – thematic analysis of the transcripts Preplacement questionnaire based on prior experience in RACF and placement expectations/attitudes Pre and post placement questionnaire – Dementia knowledge using the Dementia Knowledge Assessment Tool Version 2 (D-KAT2)	The qualitative data findings are presented under the overarching categories of ‘Students’ perceptions of beneficial learning experiences in the RACFs’, ‘Interacting with residents in the RACFs’ and ‘Working with residents requiring a palliative approach to care’ Preplacement questionnaire showed 2/3 had had some interaction with a RACF before, but most had not worked in one. Half responded neutrally to the prospect of engaging in a residential aged care placement, almost half (n = 9, 43%) were ‘unhappy’ about the prospect of the placement in general The mean score for the sum of total correct D-KAT2 answers at pre-test was 16.53 (SD = 2.96) out of a possible score of 21, and at post-test 18.65 (SD = 2.00)	Limitations: Sample size Geographic location – only one state used in sample Conclusion: During the 5-day placement, the students interacted with a range of aged care residents, including older people with dementia, developing key communication and interpersonal skills, and an understanding of the complexities of care regarding older people with life-limiting conditions
Ross, L., & Williams, B. (2014). Paramedics developing rapport with the elderly: a pilot study. *Journal of Paramedic Practice*, 6(3), 128–136	1) To investigate whether paramedic students’ active engagement with ‘real elderly patients’ 2) To investigate the impact self-reflection will have on paramedic students’ development of rapport with the elderly	11, 2nd year paramedic students at an Australian university + 11 independently living elderly residents	This pilot study utilised mixed methods incorporating an interventional study design with focus groups Pre and post activity rapport questionnaire—participants to rate perceptions of themselves across the 7 traits: confident, empathetic, humane, personal, frank, respectful and thorough, across a five-point Likert scale Focus group for the students and the elderly participants – thematic analysis of the transcripts	Revealed three items to be statistically significant and have a large effect size: Confident (*p* < 0.01, r = 0.55), Empathetic (*p* = 0.03, r = 0.48) and Overall ability to develop rapport with the elderly (*p* < 0.01, r = 0.58). Two items trended positively with medium effect size; Humane (*p* = 0.16, r = 0.30) and Thorough (*p* = 0.08, r = 0.37) Themes emerging from the students – Assertiveness, communication, attitudes, rapport, and transferable skills Themes emerging from the elderly participants – Respect, Communication, empathy, rapport, and transferable clinical skills	Limitations: Selection bias Small sample size Self-report data from the RQ was not necessarily indicative of improvement in rapport building skills, RQ not psychometrically tested Conclusion: Student’s recorded improved confidence, empathy and overall ability to build rapport with older people through this engagement activity. In addition, students who received guided self-reflection displayed greater improvements in the above three areas than their control counterparts
Ross, L. J., Jennings, P. A., Gosling, C. M., & Williams, B. (2018). Experiential education enhancing paramedic perspective and interpersonal communication with older patients: a controlled study. *BMC medical education*, 18(1), 239	The aim was to determine the effects of an educational intervention with older people on student paramedic’s knowledge, attitudes, and behaviour toward older patients	124, 2nd year paramedic students at an Australian university	Controlled before and after study Three instruments were used to collect data from the participants at Time 1 (pre-intervention Feb 2017), and Time 2 (post-intervention May 2017) 1. Aging Semantic Differential (ASD) 2. Facts on Aging Quiz 2 (FAQ2) 3. Kalamazoo Communication Skills Assessment (KCSA) is a modified version of the original Kalamazoo Essential Elements Communication Checklist. KCSA was completed by the student, the patient and a clinician following a 10 min patient-centred interview with an older adult at both Time 1 and Time 2	ASD—Both groups displayed slightly positive attitudes toward older adults at Time 1 prior to the intervention. At Time 2 both groups had a slight decrease in attitudes, while they remained on the positive side of neutral. Neither change was statistically significant; *p* = 0.12 and *p* = 0.58 respectively FAQ2 – There was little difference found between the intervention and control groups in FAQ2 scores prior to the intervention. The intervention group scores improved from Time 1, (mean ± SD: 10.1 ± 1.94) to Time 2, (mean ± SD: 10.5 ± 2.06), however this was not statistically significant (*p* = 0.51), with a small effect size (η^2^ = 0.01) The total KCSA mean score for the intervention group clinician rating improved by 2.8 from Time 1, (mean ± SD: 15.4 ± 3.09) to Time 2, (mean ± SD: 18.2 ± 3.20). This was statistically significant (*p* < 0.001), with a large effect size (η^2^ = 0.41). Similarly, the control group clinical rating improved by 2.9 from Time 1, (mean ± SD: 16.2 ± 2.01) to Time 2, (mean ± SD: 19.1 ± 3.60). This was also statistically significant (*p* < 0.001), with a large effect size (η^2^ = 0.35)	Limitations: Not fully randomised, but groups appeared homogeneous No control over the student’s prior experience with older patients No control over students discussing the intervention, with the control group Conclusion: This study affirms that paramedic students have poor knowledge and slightly positive attitudes toward older patients As the first study to observe, measure and report on the interpersonal communication skills of paramedic student’s with ‘real’ older patients we can report that these skills were fair—good at baseline and improved to good—very good post the intervention Overall improvement was notably better in the ‘understanding the patients perspective element’ for the intervention group who had conducted one–one visits with an older person
Furseth, P. A., Taylor, B., & Kim, S. C. (2016). Impact of Interprofessional Education Among Nursing and Paramedic Students. *Nurse Educ*, 41(2), 75–79	The aim was to compare the effects, among nursing and paramedic students, of 2 different types of simulation sessions on attitudes toward IPE and health care teams, along with student satisfaction and self-confidence	131 nursing and 58 paramedic students at a University in the USA	Pretest and posttest design 1.Attitudes Toward IPE: The 15-item assesses students’ perception of effectiveness of teamwork and collaboration among professionals in the IPE setting (5-point, Likert-type responses, ranging from 1 (‘‘strongly disagree’’) to 5 (‘‘strongly agree’’)) 2.Attitudes Toward Interprofessional Healthcare Teams (IPT): 14 items and uses 5-point, Likert-type responses ranging from 1 (‘‘strongly disagree’’) to 5 (‘‘strongly agree’’), rating whether working with other health care professionals would improve the quality of patient care 3.Student Satisfaction and Self-confidence in Learning: 2 subscales with 5-point, Likert-type responses ranging from 1 (‘‘strongly disagree’’) to 5 (‘‘strongly agree’’)	Nursing students: Attitudes toward IPT, the mean score change from pretest to posttest was significantly greater after the handoff communication sessions in comparison with the MCI sessions (+ 0.33 vs 0, respectively; PG 0.001) Attitudes toward IPE, the mean score change was significantly greater after handoff communication sessions in comparison with the MCI sessions (+ 0.23 vs j0.08, respectively; *p* = 0.001) Paramedic students: There was little change Nursing students: Satisfaction score was significantly higher after the handoff communication sessions in comparison with the MCI sessions (4.38 vs 3.87, respectively; PG 0.001), and the mean self-confidence score was also higher after the handoff communication sessions in comparison with the MCI sessions (4.08 vs 3.61, respectively; PG 0.001) Paramedic students: Although the mean scores for satisfaction and self-confidence among the paramedic students were generally higher than those of the nursing students, no significant differences in mean satisfaction score (4.45 vs 4.48; *p* = 0.809) and mean self-confidence score (4.55 vs 4.43; *p* = 0.528) were found between handoff communication and MCI simulation sessions	Limitations: Paramedic students participated in both activities whereas the nurses did not, meaning they had more exposure to IPE Nursing students were predominantly female and paramedic student male Conclusion: Simulation-based IPE sessions appears to have had a significant impact on nursing students’ attitudes toward IPE and IPT Self-confidence after participating in the IPE handover activity were higher than that of the MCI activity
Johnston, T., MacQuarrie, A. J., & Rae, J. (2014). Bridging the gap: Reflections on teaching interprofessional communication to undergraduate paramedic and nursing students. *Australasian Journal of Paramedicine*, 11(4), 1–9	To address and remove previously identified barriers to IPE and explore how simulation might be used to improve interprofessional communication between and among our paramedic and nursing students, especially during patient handover	130, 3rd year paramedic and 70 2nd year nursing students at an Australian university	Semi-structured evaluation using prompting questions for both students, academics, and all staff involved Responses were grouped and analysed to generate themes	Themes relating to interprofessional communication and IPE Data related to these questions: What worked well? What did not work well? What stumped you? What would you do differently next time?	Limitations: Did not use or measure against a validated communication tool in order to quantify the effectiveness of using IPE to improve patient handover Did not administer a pre-project survey and the feedback we elicited from participants was only obtained post-exercise and combined Conclusion: Reports from our students confirm that the even this simulated experience can be daunting. It is possible for academics to work across disciplines to prepare students to meet these pressures, and IPE through simulation is a useful method
Ford, R., Webb, H., Allen-Craig, S., Goodwin, V., D'Antonio, J., & Lofts, C. (2014). A simulated wilderness exercise: the development of relational competence in paramedic students. *Journal of Paramedic Practice*, 6(11), 574–583	The aim of this study was to assess the impact of a cognitively and physically challenging SWE on the development of clinical and relational competence in senior paramedic students	29 senior students enrolled in the nursing/paramedicine double degree at an Australian university	Qualitative data gathered via: 1. field diary: Reflective journaling, that is, to record what they noticed/experienced (discuss feelings and emotions) to make sense (analyse and evaluate) and to make meaning (action plan for future development) 2. focus groups: Conducted over 60 min and addressed a schedule of questions designed to elicit responses on key study objective Thematic analysis by whole team	Emerging theme was ‘new understandings’ and the sub-themes included: Interpersonal relationships Maturity, respect and tolerance Self-awareness within the team environment Belonging and professional identity	No limitations Conclusion: The SWE offered paramedic students the opportunity for new learning in relational competence, a competence seen as essential for a positive team result in paramedics’ often high-risk working environment. There is an important practice implication from our study findings, namely, our graduates will transition to on-road paramedic work with greater ease. They will bring a high level of understanding about communication strategies and team cohesion
Ross, L., & Bertucci, J. (2014). Pathway to Paramedicine Program Perspectives – Part 1 Paramedic Students. *Australasian Journal of Paramedicine*, 11(6)	The aim was to evaluate the student paramedics’ perspectives of the Pathway to Paramedicine Program	14, 2^nd^ year paramedic students enrolled in either paramedicine or the nursing paramedicine double degree at an Australian university	Intervention study: Pathway to Paramedicine Evaluation (PPE) survey was developed by the authors. It includes 12 questions utilising a 5-point Likert scale with 1 (strongly disagree) to 5 (strongly agree) and two free text questions Thematic analysis of the open-ended questions by both authors	All 12 Likert scale questions on the PPE achieve a median score of 4 or higher. Four items achieved a maximum median of 5 (strongly agree) Themes emerging from open-ended questions included: The program helped students reinforce their knowledge and skills The program had a good ratio of students to mentors The paramedic students enjoyed and benefited from the teacher/mentor experience	Limitations: Small sample Conclusion: This pilot study showed that the paramedic students had an overwhelmingly positive perspective of the Pathway to Paramedic Program. The paramedic students felt the program helped develop their interpersonal communication skills and reinforced their clinical skills and knowledge
Ross, L. (2012). Interpersonal skills education for undergraduate nurses and paramedics. *Journal of Paramedic Practice*, 4(11), 655–661	This review aims to determine what teaching techniques/methods are being used to teach undergraduate nurses’ and paramedics’ interpersonal skills and how effective they are	6 relevant articles found via inclusion and exclusion methods	Literature review Key terms used in 4 databases (Medline, EMBASE, PsycINFO, and ERIC), limited to post 2000, with relevance to the question were included in this study 2838 articles found, and abstracts reviewed, and duplicates removed, produced 64. This was further reduced to 6 by using PIO inclusion criteria	Theme emerging in articles: Online discussion methods Service-learning methods Audio tapes method Role play method	No limitations Conclusion: Review has highlighted the need for undergraduate programmes to place more emphasis on the teaching and learning of interpersonal skills in order to better prepare student nurses and paramedics for the requirements of their profession. In addition, demonstrated a wide array of teaching strategies can be employed to achieve this. Best methods appeared to use a combination of teaching strategies, and engaged the students in an activity which had as much realism as possible
Twinley, R. (2012). Developing communication skills in occupational therapy, and paramedic students. *Journal of Paramedic Practice*, 4(12), 705–714	Explored how Allied Health Professional (AHP) students are taught communication skills, and a need for research that evaluates the student opinion of learning communication skills	44 occupational therapy students and 11, 3rd year paramedic students at a University in the United kingdom	Action research: Mixed method questionnaire Questionnaires comprised of 34 attitudinal scaled responses under the headings: ‘your programme’; ‘verbal communication skills’; ‘written communication skills’; ‘non-verbal communication skills’ and ‘preparation for placements and practice’. There were two open-ended questions to gain answers in the respondents’ own words. Closed answered questions involved a Likert scale with 1 (strongly disagree) to 5 (strongly agree) Analysis of open-ended questions was conducted	Prioritising communication skills: Majority listed listening skills Student recommendations: More practical sessions Expectations: Majority agreed or strongly agreed that they expected to be taught communication skills and that they were Verbal and written communication skills: Students understand how they are assessed, and they know they require this skill to be a competent HCP Non-verbal communication skills: Differing results here showing that 20% of OT’s feel they are not taught this. Both groups would welcome more feedback with this skill Preparation for placements and practice: Both groups show the students feeling less prepared for their first placement, then increasingly more prepared	Limitations: Suggestion of researcher bias Small sample Questionnaire – low response rate possibly as it was sent via email; students did not complete some questions; and the use of closed questions might not allow a true opinion to be expressed Time constraints meant member-checking was not done Conclusion: Overall, findings show participants would welcome more communication skills training on their programmes. More practical sessions were also suggested to improve the student learning experience. Communication skills are seen as crucial for their own clinical practice
O’Brien, K., Moore, A., Hartley, P., & Dawson, D. (2013). Lessons about work readiness from final year paramedic students in an Australian university. *Australasian Journal of Paramedicine*, 10(4)	Objectives of the study were to investigate the perceptions of readiness for the workforce in final year paramedic at Victoria University, to understand in particular the strengths of the paramedic course and how it could be improved so that the transition into the workplace is facilitated	23 final year paramedic students at Australian universities	Mixed method survey Part 1 of our survey consisted of 64 statements about paramedic practice, subdivided into eight areas or dimensions relating to paramedic practice Participants were required to choose from six possible responses in relation to how well their degree had prepared them with respect to each specific topic: very inadequately [1]; inadequately [2]; somewhat inadequately [3]; somewhat adequately [4]; adequately [5]; and very adequately [6] Part 2 of the survey consisted of five open- ended questions relating to the transition from student to paramedic practitioner and critique of the paramedic course Use of NVivo analysis the qual data	Somewhat adequately and adequately for all 8 dimensions, with ethics & legal responsibilities, clinical skills and practical skills scoring the highest Preparedness for transition into the paramedic workforce: 2/3 feeling prepared, but some students considering they will learn more once they are in the industry or have more experience Best aspects of the course: Practical and placement elements Placements: Majority found them useful with other stating they would like more placement time Course Improvements: More varied placements and more practical time	Limitations: Self-selection bias Voluntary based sample Timing of the survey may have limited the number of students prepared to take part Conclusion: Results of this survey suggest students perceive themselves to be ‘somewhat prepared’ or ‘prepared’ for entry into the workforce Clinical practice opportunities are valued however, a common theme emerging was a desire for more and varied clinical placements
Włoszczak-Szubzda, A., Jarosz, M. J., & Goniewicz, M. (2013). Professional communication competences of paramedics—practical and educational perspectives. *annals of agricultural and environments medicine*, 20(2), 366–372	Study explored the level of individual communication competences of paramedics (knowledge, motivation, and skills)? Whilst exploring differences in the level of professional communication competences according to the model of education?	31 occupationally active paramedics 54 paramedic students with standard undergraduate program 20 students with extra communication education 20 paramedic institutions across Poland	1. analysis of documentation (standards, education schedules, curricula and syllabuses)—20 paramedic schools in Poland, which were available on the websites of these educational facilities 2. diagnostic survey concerning professional communication competences of paramedics – self-designed questionnaire 3. testing of professional self-evaluation from the paramedic’s aspect – the 20 items adjective check list The 54 items in the questionnaire and test examine scope of motivation, scope of knowledge, and scope of skills in relation to communication competence	Analysis of documentation: Limited occupational specific communication skills taught Diagnostic survey: Communication skills higher in those students who had conducted extra education Professional self-evaluation: Communication skills higher in those students who had conducted extra education	No limitations Conclusion: The efficiency of shaping communication competences among paramedic students, based on education within the scope of general psychology only, is relatively low Certain communication competences acquired during undergraduate education are subject to regression during occupational activity, and due to occupational stress, are replaced by undesired defence mechanisms, such as psychological resistance or withdrawal
